# Evaluation of the relationship between the level of addiction and exhaled carbon monoxide levels with neutrophil-to-lymphocyte and platelet-to-lymphocyte ratios in smokers

**DOI:** 10.18332/tid/149227

**Published:** 2022-06-17

**Authors:** Melih Güden, Sibel Tunç Karaman, Okcan Basat

**Affiliations:** 1 Department of Family Medicine, Gaziosmanpasa Training and Research Hospital, University of Health Sciences, Istanbul, Turkey

**Keywords:** exhaled carbon monoxide, inflammation, neutrophil/lymphocyte ratio, platelet/lymphocyte ratio, smoking

## Abstract

**INTRODUCTION:**

Smoking has been reported to increase systemic inflammation. The neutrophil-to-lymphocyte ratio (NLR) and platelet-to-lymphocyte ratio (PLR) are used as markers for systemic inflammation. In this study, the primary aim was to determine the NLR and PLR ratios in smokers. Secondly, we aimed to evaluate the relationship between the level of addiction and carbon monoxide (CO) level in the expiratory air, with these ratios.

**METHODS:**

This study was designed as a single-center, cross-sectional study. It was conducted with chronic smokers aged 18–40 years, without known health problems, visiting the smoking cessation outpatient clinic of a tertiary hospital. Sociodemographic data and smoking characteristics were collected, and exhaled CO levels were measured. Complete blood count (CBC) results were recorded, including NLR and PLR.

**RESULTS:**

The mean age of 247 patients was 31.2±6.1 years, with the majority of patients (68.4%) being male. While the mean value of CO was 11.6±5.6 ppm, 42.1% of cases had a high level of addiction. A statistically significant relationship was found between NLR and addiction levels, the CO level, and the amount of smoking in cigarettes/day and packs/year (all p=0.000). A statistically significant relationship was also found between PLR and addiction levels, CO level, cigarettes/day and packs/year (p=0.000, p=0.03, p=0.000, p=0.003, respectively).

**CONCLUSIONS:**

We found that as the level of addiction, cigarette use, and exhaled CO levels increased in smokers, NLR and PLR increased. Our data revealed that NLR and PLR may be a simple and easily assessable proxy of systemic inflammation in smokers.

## INTRODUCTION

Smoking causes many severe diseases and deaths and has been defined as the most critical public health problem by the World Health Organization (WHO)^1^. There are thousands of substances in cigarette smoke that are pharmacologically active, antigenic, carcinogenic, and addictive, including nicotine and carbon monoxide (CO)^2^. Through these substances, it is known that cigarette smoke causes damage to the airway epithelium, activates cytokines and chemokines, increases vascular and intracellular adhesion molecules, and triggers inflammation. Besides, it has been suggested that smoking disrupts the vasodilation in the vascular endothelium and causes endothelial dysfunction, which may initiate the inflammatory process^3-5^. It was found that higher tobacco consumption was associated with higher systemic inflammation in observational and genetic analyses^6^.

Many biochemical and hematological parameters can detect systemic inflammation. In the event of an inflammatory response, there is an increase in the number of neutrophils and a relative decrease in the lymphocyte count^7,8^. Platelets are known to contribute to the regulation of inflammatory reactions and to blood coagulation and hemostasis. Chronic inflammation causes thrombocytosis and relative lymphopenia^9^. The ratios of these subgroups to each other, neutrophil-to-lymphocyte ratio (NLR) and platelet-to-lymphocyte ratio (PLR), are accepted as cheap, effective, and easy to apply systemic inflammatory markers and, apart from being used for the diagnosis and determination of severity for many disease processes (cardiovascular disease, pulmonary infections, endocrine disorders, and cancers), their relationship with prognosis, morbidity and mortality has also been demonstrated^9-15^.

Several studies have shown that smoking status is associated with changes in various hematological parameters such as the neutrophil, lymphocyte, monocyte, and eosinophil counts, all of which are active in inflammation and the NLR and PLR^4,16-18^. However, to the best of our knowledge, the relation between addiction levels, exhaled carbon monoxide levels, and these parameters have not been studied.

In this study, the primary aim was to determine the inflammation by investigating NLR and PLR in smokers. Secondly, we aimed to evaluate the relationship between the level of nicotine addiction and carbon monoxide (CO) level in the expiratory air, with these ratios.

## METHODS

### Study design and population

This study was designed as a single-center, cross-sectional study. All participants were selected from current chronic smokers referred for the first time to the Smoking Cessation Clinic of a tertiary hospital in Turkey from January 2020 to March 2020. Those who smoked one or more cigarettes per day for at least three months were accepted as smokers. Two hundred and forty-seven people who had no known health problems, between the ages of 18 and 40 years, and agreed to participate, were included in the study. According to the G-power analysis made in line with the reference studies, the minimum number of participants required for the study was 146 with a 95% CI.

### Exclusion criteria

Since inflammatory markers could be affected, patients under the age of 18 and over 40 years, those with known anemia, leukocytosis or leukopenia, chronic alcohol addiction, substance abuse, malignancy, a history of upper respiratory tract infection in the last three weeks, non-steroidal anti-inflammatory drugs (NSAID) use up to 1 week ago, steroid use within last six months, those with diabetes mellitus, hypertension, hypo-hyperthyroidism, kidney failure, chronic liver disease, asthma, COPD (chronic obstructive pulmonary disease), ischemic heart disease, heart failure, chronic inflammation, and pregnant women were excluded from the study.

### Data collection tools

*Patient information form*

A patient information form was formulated, which included the participants’ sociodemographic characteristics (age, gender), smoking status (cigarettes/day and packs/year), medical history, BMI (body mass index, kg/m^2^), exhaled CO measurement (ppm) and complete blood count (CBC) parameters. Packs/year were calculated as the number of cigarettes smoked per day multiplied by the number of years smoked. Patients were subgrouped according to BMI as: underweight <18.5; normal weight 18.5–24.9; overweight 25–29.9; obese 30.0–39.9 ; and morbidly obese ≥40 kg/m^2^.

*Fagerström test for nicotine dependence (FTND)*

The FTND^19^, for which the Turkish validity and reliability study was conducted by Uysal et al.^20^ in 2004, was used to determine the degree of participants’ nicotine dependence. The dependence level was categorized according to the following FTND scores: 0–3 low; 4–7 moderate; and 8–10 high.

*Exhaled CO measurement*

Exhaled CO increases in smokers and is a biomarker frequently used in the diagnosis, treatment, and follow-up stages of cigarette dependence^21^. Measurements of all participants were made at least 1 hour after smoking their last cigarette. Exhaled CO measurements were performed by authorized healthcare staff using piCO + Smokerlyzer (Bedford Scientific, Maidstone, UK, 2016) devices. The participants were asked to hold their breath for 20 seconds and then slowly blow into the Smokerlyzer mouthpiece. The researcher stayed with the participants and instructed them about the protocol at the time of their attendance for the study.

*Blood examination and evaluated parameters*

The ante-cubital vein was used to obtain CBC samples following 12 hours of fasting. CBC parameters including white blood cells (WBC) subgroups (neutrophil, lymphocyte, eosinophil and monocyte counts) and platelet counts were recorded. NLR, PLR and eosinophil-to-lymphocyte ratio (ELR) values were calculated by dividing the absolute neutrophil, platelet and eosinophil counts by the absolute lymphocyte count, respectively. In addition, lymphocyte-to-monocyte ratio (LMR) values were calculated by dividing the absolute lymphocyte counts by the absolute monocyte count.

### Statistical analysis

The IBM SPSS Statistics 22 program was used for statistical analysis. The compliance of the parameters to normal distribution was evaluated with the Shapiro-Wilk test. In addition to descriptive statistical methods (mean, standard deviation, frequency) in more than two-group comparisons, one-way ANOVA was used when numerical variables showed normal distribution, and the Kruskal–Wallis test was used when there was no normal distribution. Mann–Whitney U test was used to determine the group that caused the difference. Mann–Whitney U test evaluated the comparisons of normally distributed parameters between two groups. Pearson’s correlation analysis was performed to examine the relationships between parameters that conform to a normal distribution, and Spearman’s rho correlation analysis was used to examine relationships between parameters that did not conform to a normal distribution. A p<0.05 was considered statistically significant.

## RESULTS

A total of 247 smokers were included in the study. The participants’ mean age was 31.2±6.1 years, and 68.4% were male (n=169). The mean number of cigarettes smoked was 27.3±10.9 cigarettes/day and 19.7±12.0 packs/year. The mean value of exhaled CO was 11.6±5.6 ppm. The mean FTND score was 6.6±2.3 (range: 1–10), and 42.1% of the participants were highly dependent. The mean BMI was 25.4±3.3 kg/m^2^ (range: 16.5–34.9). Table 1 presents the data on the sociodemographic and smoking characteristics of the participants. Table 2 shows the data regarding the participants’ CBC parameters, and NLR, PLR, ELR and LMR values.

**Table 1:** Evaluation of sociodemographic and smoking characteristics of the study population (N=247)

*Characteristics*	*Range*	*Mean±SD (median)*
**Age** (years)	18–40	31.2±6.1
**BMI** (kg/m^2^)	16.5–34.9	25.4±3.3
**Smoking amount** (pieces/day)	4–60	27.3±10.9 (25)
**Smoking amount** (packs/year)	1–52	19.7±12.0 (17)
**Age when began smoking** (years)	8–35	16.4±3.5
**Total years of smoking**	2–29	14.7±6.3
**Exhaled CO level** (ppm)	1–32	11.6±5.6
**FTND score**	1–10	6.6±2.3 (7)
	***n***	***%***
**Gender**		
Female	78	31.6
Male	169	68.4
**Level of dependence**		
Low	26	10.5
Medium	117	47.4
High	104	42.1

BMI: body mass index (kg/m^2^). CO: carbon monoxide (ppm). FTND: Fagerström test for nicotine dependence.

**Table 2:** Distribution of the data on the complete blood count parameters of the participants (N=247)

	*Range*	*Mean±SD (median)*
**WBC** (10^3^/mm^3^)	5.3–10.9	8.5±1.5
**RBC** (10^6^/mm^3^)	3.7–5.9	5.0±0.5
**RDW** (%)	11.9–17.7	13.3±0.9
**HB** (g/dL)	12–17.8	15.2±1.4
**HCT** (%)	35.7–52.8	44.8±3.9
**MCV** (fL)	76.8–100	90.7±4.2
**Platelet** (10^3^/mm^3^)	151–450	257.4±55.1
**MPV** (fL)	7.6–14	9.7±1.1
**Neutrophil** (10^3^/mm^3^)	2.3–8.2	5.0±1.3
**Eosinophil** (10^3^/mm^3^)	0.0–1.1	0.2±0.2
**Lymphocyte** (10^3^/mm^3^)	1.2–4.7	2.7±0.7
**Monocyte** (10^3^/mm^3^)	0.1–1.2	0.5±0.2
**NLR** (%)	0.8–5.7	2.0±0.7 (1.9)
**PLR** (%)	39.1–275.7	102.1±31.0 (97.9)
**LMR** (%)	1.7–12.4	5.3±1.7 (5)
**ELR** (%)	0–0.5	0.1±0.0 (0.1)

WBC: white blood cells. RBC: red blood cells. RDW: red cell distribution width. HB: hemoglobin. HCT: hematocrit. MCV: mean corpuscular volume. MPV: mean platelet volume. NLR: neutrophil-to-lymphocyte ratio. PLR: platelet-to-lymphocyte ratio. LMR: lymphocyte-to-monocyte ratio. ELR: eosinophil-to-lymphocyte ratio.

**Figure 1:**
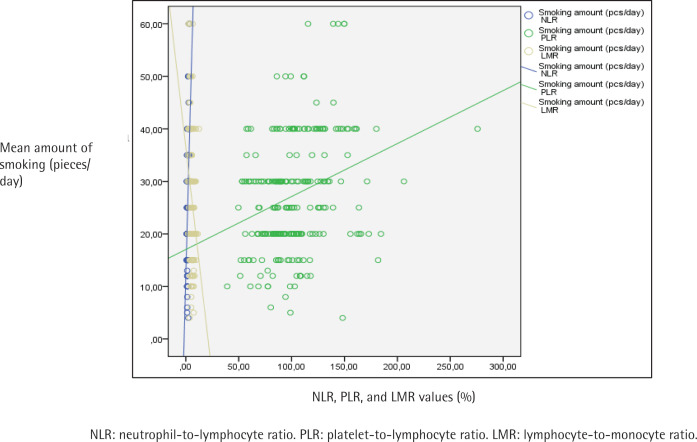
The relationship between participants’ NLR, PLR and LMR values, and the mean amount of smoking (pieces/day)

**Figure 2:**
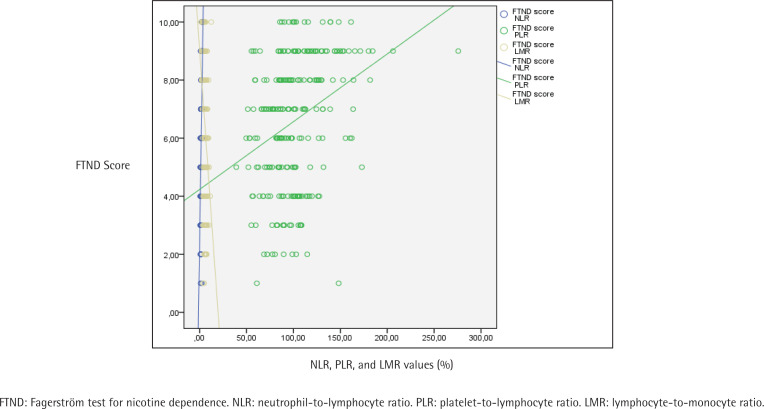
The relationship between participants’ NLR, PLR, and LMR values, and FTND score

**Figure 3:**
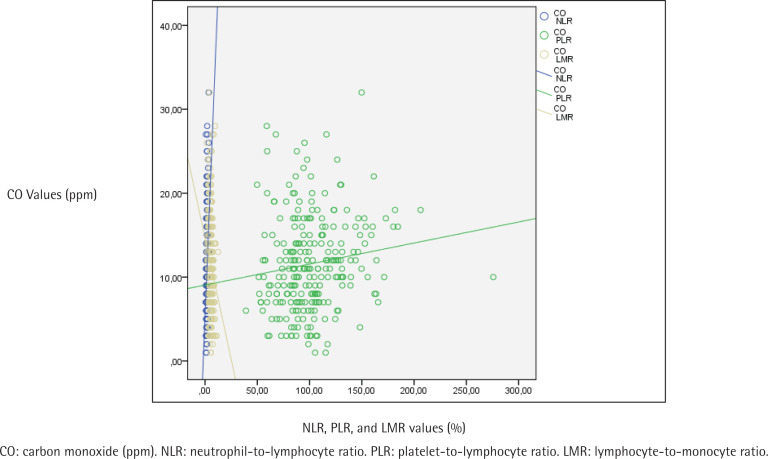
The relationship between participants’ NLR, PLR, and LMR values, and exhaled CO values

Table 3 displays the evaluation of CBC parameters, NLR, PLR, LMR, ELR and exhaled CO values according to addiction levels of the participants. A statistically significant difference was found between addiction levels and NLR values (p<0.001). When the paired comparisons were made to determine the difference, the NLR values of those with a high level of addiction were statistically significantly higher than the others (p<0.001). The NLR values of those with medium level of addiction were found to be statistically significantly higher than those with low level of addiction (p=0.003) (Table 3). A statistically significant difference was found between addiction levels and PLR values as well (p<0.001). The paired comparisons performed to detect the difference determined that PLR values of those with a high level of addiction were found to be statistically significantly higher than those with low and medium levels (p<0.001). No statistically significant difference was found in PLR values between the groups with low and medium FTND scores (Table 3). A statistically significant difference was found between addiction levels and LMR values (p<0.001). As a result of the paired comparisons made to detect the difference, LMR values of those with a high level of addiction were found to be statistically significantly lower than those with low and medium level of addiction (p<0.001) (Table 3). Also, the relationship between NLR, PLR and LMR values and the mean amount of smoking (cigarettes/day), FTND score, and exhaled CO values are shown in Figures 1, 2 and 3, respectively.

**Table 3:** Evaluation of participants’ complete blood count parameters, NLR, PLR, LMR, ELR and CO values according to their addiction levels (N=247)

	*Level of dependence*			*p*
	*Low*	*Medium*	*High*	
	*Mean±SD (median)*	*Mean±SD (median)*	*Mean±SD (median)*	
**WBC** (10^3^/mm^3^)	7.8±1.4	8.3±1.5	8.8±1.4	0.001^a^*
**RBC** (10^6^/mm^3^)	4.8±0.4	5.0±0.4	5.0±0.5	0.03^a^*
**RDW** (%)	13.1±0.6	13.2±1.1	13.3±0.8	0.12^b^
**HB** (g/dL)	14.6±1.4	15.2±1.4	15.3±1.4	0.05^a^
**HCT** (%)	43.4±3.6	44.7±3.9	45.4±4.0	0.04^a^*
**MCV** (fL)	91.4±4.3	90.5±4.5	90.8±3.9	0.59^a^
**PLT** (10^3^/mm^3^)	264.8±55.3	257.6±56.8	255.2±53.6	0.73^a^
**MPV** (fL)	9.3±0.8	9.7±1.1	9.7±1.1	0.11^a^
**Neutrophil** (10^3^/mm^3^)	4.0±0.9	4.6±1.1	5.8±1.1	<0.001^a^*
**Eosinophil** (10^3^/mm^3^)	0.3±0.2	0.2±0.2	0.2±0.2	0.05^b^
**Lymphocyte** (10^3^/mm^3^)	3.0±0.5	2.9±0.7	2.3±0.6	<0.001^a^*
**Monocyte** (10^3^/mm^3^)	0.5±0.1	0.5±0.1	0.6±0.2	0.21^a^
**NLR** (%)	1.4±0.3 (1.4)	1.7±0.5 (1.6)	2.6±0.7 (2.5)	<0.001^b^*
**PLR** (%)	90.5±20.2 (89.6)	92.9±25.5 (88.8)	115.2±34.1 (111.3)	<0.001^b^*
**LMR** (%)	6.2±1.4 (6.4)	5.7±1.6 (5.4)	4.5±1.6 (4.2)	<0.001^b^*
**ELR** (%)	0.1±0.1 (0.1)	0.1±0.1 (0.1)	0.1±0.1 (0.1)	0.63^b^
**Exhaled CO** (ppm)	7.6±3.4	9.5±4.7	15.0±5.2	<0.001^a^*

a One-way Anova test.

b Kruskal–Wallis test.

* p<0.05.

WBC: white blood cells. RBC: red blood cells. RDW: red cell distribution width. HB: hemoglobin. HCT: hematocrit. MCV: mean corpuscular volume. MPV: mean platelet volume. NLR: neutrophil-to-lymphocyte ratio. PLR: platelet-to-lymphocyte ratio. LMR: lymphocyte-to-monocyte ratio. ELR: eosinophil-to-lymphocyte ratio. CO: Carbon monoxide (ppm).

As indicated in Supplementary file Table 1, no statistically significant difference was found between males and females in terms of NLR and PLR parameters. However, a statistically significant difference was found between the levels of addiction in women and men in terms of NLR and PLR values (p=0.001 for both females and males). When the paired comparisons were made to determine the difference, the NLR and PLR values of those with a high level of addiction in women were found to be statistically significantly higher than those with a low and medium level of addiction (p=0.001, p=0.001 for NLR; p=0.037, p=0.001 for PLR). Similarly, the NLR and PLR values of those with a high level of addiction in men were found to be statistically significantly higher than those with low and medium addiction (p=0.001, p=0.001 for NLR; p=0.004, p=0.001 for PLR). There was no statistically significant difference between those with low and medium addiction in both genders (p>0.05; both for NLR and PLR) (Supplementary file Table 1).

LMR values of males were found to be statistically significantly higher than for females (p=0.02). ELR values of males were found to be statistically significantly lower than for females (p=0.006).

As Table 4 demonstrates, no statistically significant correlation was found between age, age of smoking onset, and BMI, with NLR, PLR and LMR values. An inverse, statistically significant correlation was found between age, age of smoking onset, with BMI and ELR (p=0.04, p=0.03, p=0.03, respectively). A positive correlation was found between the exhaled CO value with NLR and PLR values (p<0.001, p=0.03, respectively), and an inverse statistically significant correlation was found with the LMR (p=0.005). No statistically significant relationship was found between the total smoking time and the values of NLR, PLR, LMR and ELR parameters (p>0.05). A positive correlation was found between the number of cigarettes smoked per day and NLR and PLR (both p<0.001) and a negative statistically significant correlation with LMR (p<0.001). A positive and an inverse, statistically significant correlation was found between the amount of cigarette use in packs/year and NLR and PLR values (p<0.001, p=0.003, respectively). A positive correlation was found between the FTND score and NLR and PLR, and an inverse statistically significant relationship with LMR (p<0.001) (Table 4).

**Table 4:** Evaluation of the correlation between study parameters and NLR, PLR, LMR and ELR values of the participants (N=247)

		*Age*	*Age at smoking onset*	*Total years of smoking*	*Smoking amount (pieces/day)*	*Smoking amount (packs/year)*	*FTND score*	*CO*	*BMI*
NLR	r	0.08	-0.01	0.12	0.53^†^	0.41^†^	0.74^†^	0.40	-0.01
	p	0.16	0.77	0.05	<0.001*	<0.001*	<0.001*	<0.001*	0.85
PLR	r	0.04	0.03	0.06	0.28^†^	0.18^†^	0.33^†^	0.13	-0.03
	p	0.45	0.57	0.31	<0.001*	0.003*	<0.001*	0.03*	0.56
LMR	r	0.04	0.03	0.01	-0.32^†^	-0.14^†^	-0.39^†^	-0.17	0.03
	p	0.44	0.61	0.86	<0.001*	0.022*	<0.001*	0.005*	0.55
ELR	r	-0.02	-0.01	0.005	0.06^†^	0.01^†^	0.02^†^	0.06	0.09
	p	0.04*	0.03*	0.93	0.29	0.86	0.74	0.31	0.03*

Pearson correlation analysis.

† Spearman rho correlation analysis.

* p<0.05. NLR: neutrophil-to-lymphocyte ratio.

PLR: platelet-to-lymphocyte ratio. LMR: lymphocyte-to-monocyte ratio. ELR: eosinophil-to-lymphocyte ratio.

Finally, as shown in Supplementary file Table 2, there was no statistically significant correlation between BMI groups and NLR and PLR according to dependence levels (p>0.05) (Supplementary file Table 2).

## DISCUSSION

Our results demonstrate once again the relationship between smoking and inflammation through the NLR and PLR, similarly to previous studies^16,17,22-24^. As a contribution to the literature, in addition to previous studies evaluating the relationship with the amount of consumption, it has been shown that the NLR and PLR in smokers increase as the level of addiction and exhaled CO levels increase. Therefore, it is thought that they can be used as a marker to predict chronic inflammation and chronic diseases caused by smoking.

There are various biomarkers that have been used to detect systemic inflammation. Due to the changes caused by the inflammation in neutrophils, platelets, and lymphocytes, NLR and PLR have been determined as inflammatory markers. In general, a high NLR is associated with a high mortality rate and a poor prognosis for a condition or a disease^25^. Different values of NLR in different populations (smokers or no-smokers, people with cancer or with any other specific chronic condition) are cited in the literature. However, no universal cut-off value has been proposed currently on the basis of reference values coming from a healthy population^8^. In one of the studies conducted to determine the cut-off, Forget et al.^8^ found normal NLR values of non-geriatric adults in good health between 0.8 and 3.5, with a mean of 1.7±2.0. However, since it was a retrospective study, no information could be obtained about smoking and possible chronic conditions^8^. Aydın et al.^7^, on the other hand, aimed to define the reference ranges of NLR values in different gender and age groups and to investigate the differences between the groups. It has been observed that after NLR values increased progressively until the twenties, they entered a plateau period and tended to rise again after the age of 60 years.

As is known, smoking causes chronic systemic inflammation, even in individuals without comorbidities. There are many studies on the effect of smoking on hematological parameters. In some previous studies, the relationship between smoking and NLR and PLR has been directly investigated^16,17,22-24^.

In a retrospective study conducted by Tulgar et al.16, the mean NLR value was 2.1±0.9 in smokers and 1.7±0.6 in non-smokers. This elevation found in smokers was statistically significant. When smokers were divided into groups according to the amount of consumption, it was found that the NLR value increased in direct proportion to the number of packs/year. In a study conducted by Gümüş et al.^17^, the NLR values of smokers and non-smokers were compared, and it was found to be statistically significantly higher in smokers (2.1±1.4 in smokers, 1.9±0.8 in non-smokers). However, when the consumption of smokers in terms of packs/year and pieces/day was evaluated, no statistically significant difference was found in terms of NLR values. Kumari et al.^22^ compared hookah smokers, cigarette smokers, and non-smokers in their study, and similarly, NLR was found to be higher in smokers than non-smokers. In the study conducted by Dewi et al.^23^, where all participants were male, it was found that the higher the smoking level according to the Brinkman index, the higher the NLR and PLR values that were found. However, this relationship was not statistically significant. There was a positive linear correlation between packs/year and NLR. Çekici et al.^24^ also revealed in their study that the NLR were higher in smokers than non-smokers with a weak and positive correlation with pack-years.

Our study was similar to these studies in terms of the NLR values we found in smokers. It was also observed that the NLR values of non-smokers in the studies of Gümüş et al.^13-15,18,26,27^ and Tulgar et al.^13-15,18,26,27^, and those of smokers with low-medium addiction levels in our study were close to each other. It is thought that the NLR values of non-smokers may be affected by passive exposure to cigarette smoke, exposure to environmental/non-cigarette CO sources and/or the presence of currently unknown inflammatory conditions that may affect the NLR values. In addition, it is an expected result that the NLR value will be lower due to the relatively low number of cigarettes smoked in smokers with low-medium addiction levels. It is also possible that the content of the cigarette smoked may also affect the result. In addition to all these, the differences in the age and gender distribution of the participants in the studies may also affect the results. We would also like to point out that, unlike those studies in which smokers were compared with control groups, our study was conducted only with smokers, and an evaluation was aimed according to the addiction levels of smokers and exhaled CO levels.

In some studies, conducted with groups with different diseases such as diabetes, obesity, cancer, and COPD in the literature, the relationship of NLR with smoking was also mentioned. In the study conducted by Howard et al.^26^, which investigated the effect of sociodemographic characteristics and lifestyle on NLR in individuals, the NLR level was lower in non-smokers than in smokers at some point in their lives. In another study conducted by Fest et al.^18^, on 8715 people, NLR values were found higher among males, with advanced age, low socioeconomic status, history of cardiovascular disease, and smokers. In another study, it was found that ever smokers had a significantly higher NLR value than non-smokers^27^. In a retrospective study evaluating obese patients, the NLR value was significantly higher in obese smokers than in non-smoker obese participants^13^. In the study conducted by D’Andrea et al.^14^ with patients with non-invasive bladder cancer, the increase in NLR in smokers was found to be statistically significant. In a study conducted with COPD patients, a statistically significant positive relationship was found between the amount of cigarette consumption in packs/year and the NLR value^15^.

In our study, smokers with no known health problems were evaluated, and it was found that the NLR value increased in a statistically significant way as the addiction levels and the number of cigarettes consumed (in terms of cigarettes/day and packs/year) increased in smokers. Besides, a statistically significant relationship was found between the exhaled CO value, which is an indicator of smoking, and the NLR. Thus, similar to the results obtained in previous studies, the relationship between smoking and NLR has been proven once again, and it is thought that it can be used to predict chronic inflammation caused by smoking in clinical practice.

PLR is also a critical indicator of systemic inflammatory response and is currently used as a vital marker, especially for the onset of atherosclerosis and cardiovascular disease development^28^. Tulgar et al.^16,17^ and Gümüş et al.^16,17^ found that PLR value was lower in smokers than non-smokers in their studies. In both studies, when the consumption amounts in terms of packs/year and pieces/day were evaluated, no statistically significant difference was found in terms of PLR values. In the study of Kumari et al.^22^, PLR was found to be higher in smokers.

In our study, similar results were obtained with the study of Kumari et al.^29,30^ A positive, statistically significant relationship was found between the FTND score, addiction levels, exhaled CO level, and PLR. It is thought that risk estimation against cardiovascular diseases can be made by evaluating PLR in smokers.

To our knowledge, no studies have evaluated the relationship between exhaled CO level and NLR and PLR in the literature. However, there are few studies conducted with a patient group with CO intoxication. In these studies, the NLR value was statistically significantly higher in patients presenting with CO intoxication than the control group, and no relationship was found with PLR^29^.

In our study, which is to our knowledge, one of the first evaluating the relationship between exhaled CO level and NLR and PLR, we found a significant relationship between the value of CO measured from expiratory air and NLR and PLR, and concluded that although it was not high enough to cause intoxication, regardless of the measurement technique, CO increased the risk of inflammation. The ELR and LMR are also known as novel systemic inflammatory markers. In previous studies, it has been shown that a high ELR and a low LMR are associated with several inflammatory conditions, malignancies and mortality^31-33^. A high ELR and a low LMR are also associated with smoking^24^. In the study of Çekici et al.^24^, which aimed to investigate the effects of smoking on inflammation through ELR and LMR, the ELR were significantly higher and the LMR was significantly lower in smokers than in non-smokers. The pack-years were positively correlated with the ELR and negatively correlated with the LMR. However, their values can be affected by many factors such as gender, age, as well as clinical conditions. The results of the study of Wang et al.^34^ conducted with Chinese healthy adults, showed that LMR in males was significantly lower than in females. Similar to Wang et al.^34^, Meng et al.^35^ found the LMR value to be significantly higher in women than in men. In our study, while there was no significant difference in terms of addiction levels and smoking characteristics with ELR, an inversely statistically significant difference was found between addiction levels, smoking characteristics and LMR values like Çekici et al.^24^ In our study, unlike Wang et al.^34^ and Meng et al.^35^, LMR values were found to be statistically significantly higher in males. We also found that the ELR values in men were statistically significantly lower. However, it is seen that there is not enough data in the literature regarding the evaluation of ELR values in terms of gender.

These results support the inverse relationship between smoking and LMR, which was determined in previous studies. They are also important in terms of showing the relationship between inflammation and smoking. But, in our opinion, literature data should be enriched by different and objective studies on these markers, especially ELR.

### Limitations

Our study has several limitations. One of the limitations of this study is that a single measurement was done. Therefore, the cause of the relationship between addiction levels and NLR and PLR is unknown. Another limitation of the study is the short half-life of CO. Therefore, the measurement of exhaled CO is more related to recent exposure. In addition, although measurements of all participants were made at least 1 hour after smoking their last cigarette, we could not exclude the exposure of the participant to other, non-cigarette related CO sources. It should be noted that the measured exhaled CO level may be also affected by environmental tobacco exposure and/or non-cigarette CO sources.

## CONCLUSIONS

This study found that NLR and PLR increased in smokers as the level of dependence and exhaled CO levels increased, similar to the relationship with the amount of consumption. It is thought that determining the increase in NLR and PLR, which are essential indicators in detecting subclinical inflammation, with smoking will provide preliminary information on the development of chronic diseases related to smoking in smokers.

## Data Availability

The data supporting this research are available from the authors on reasonable request.
