# Supplementary material for: Evaluation of the relationship between the level of addiction and exhaled carbon monoxide levels with neutrophil-to-lymphocyte and platelet-to-lymphocyte ratios in smokers
Source: Tob Induc Dis. 2022 Jun 17;20:52. doi: 10.18332/tid/149227 (PMC9204713; doi:10.18332/tid/149227)
Supplement: Supplementary file 1 [file TID-20-58-s1.pdf]

SUPPLEMETARY FILE

Table-1. Evaluation of participants' NLR and PLR values according to gender and the correlation between their addiction levels and NLR and PLR values according to gender.

Gender		NLR	PLR
		**Mean±SD (median)**	**Mean±SD (median)**
	Female	2.0±0.8 (1.9)	107.7±36.4 (100.4)
	Male	2.0±0.7 (1.9)	99.4±27.9 (97.7)
	^{1}p	0.833	0.187
**Gender**	**Level of Dependence**		
Female	Low	1.5±0.4 (1.5)	98.0±24.3 (104.1)
	Medium	1.6±0.5 (1.6)	93.1±23.1 (88.8)
	High	2.6±0.8 (2.5)	128.2±43.0 (117.5)
	^{2}p	0.001*	0,001*
Male	Low	1.3±0.2 (1.4)	85.8±16.3 (89.2)
	Medium	1.7±0.5 (1.6)	92.8±26.6 (88.9)
	High	2.6±0.6 (2.6)	109.7±28.1 (105.0)
	^{2}p	0.001*	0.001*

Data presented as Mean±SD (median) values. [1]*Mann Whitney U Test* [2]*Kruskal Wallis Test.* *$*p<0.05$* NLR, neutrophil-to-lymphocyte ratio; PLR, Platelet-to-lymphocyte ratio.

Table-2: **Evaluation of the correlation between BMI groups and NLR, PLR values of the participants according to dependence levels.**

Level of Dependence	BMI	NLR Mean±SD (median)	PLR Mean±SD (median)
Low	Normal weight (n=11)	1.3±0.2 (1.3)	84.4±15.5 (80.3)
	Overweight (n=14)	1.4±0.4 (1.5)	94.7±23.3 (94.0)
	Obese (n=1)	-	-
	[1]p	0.506	0.350
Medium	Underweight (n=1)	-	-
	Normal weight (n=53)	1.6±0.5 (1.5)	92.6±24.7 (92.0)
	Overweight (n=54)	1.7±0.4 (1.7)	94.7±27.8 (89.7)
	Obese (n=9)	1.6±0.7 (1.3)	84.5±14.1 (83.5)
	[2]p	0.718	0.712
High	Underweight (n=3)	2.5±0.5 (2.6)	96.5±12.8 (90.5)
	Normal weight (n=50)	2.5±0.6 (2.5)	118.5±35.4 (115.8)
	Overweight (n=37)	2.6±0.8 (2.7)	113.0±36.7 (104.7)
	Obese (n=14)	2.6±0.6 (2.6)	113.7±24.8 (112.4)
	[2]p	0.912	0.544

Data presented as Mean±SD (median) values. [1] *Mann Whitney U Test* [2]*Kruskal Wallis test*

BMI, Body Mass Index (kg/m^2); NLR, neutrophil-to-lymphocyte ratio; PLR, Platelet-to-lymphocyte ratio.

© 2022 Güden M. et al.
